# The stimulation and inhibition of beta-2 adrenergic receptor on the inflammatory responses of ovary and immune system in the aged laying hens

**DOI:** 10.1186/s12917-021-02892-z

**Published:** 2021-05-22

**Authors:** Ali Hatefi, Ahmad Zare Shahneh, Zarbakht Ansari Pirsaraie, Ali Mohammad Alizadeh, Mohammad Pouya Atashnak, Reza Masoudi, Frederic Pio

**Affiliations:** 1 Department of Animal Science, University of Tehran, Karaj, Iran; 2 Department of Animal Science, Sari Agricultural and Natural Resources University, Sari, Iran; 3 Cancer Research Center, Tehran University of Medical Sciences, Tehran, Iran; 4 Molecular Biology and Biochemistry Department, Simon Fraser University, Burnaby, British Columbia Canada; 5 Animal Science Research Institute of Iran (ASRI), Agricultural Research Education and Extension Organization (AREEO), Karaj, Iran

**Keywords:** Immune function, Laying hen, Propranolol, Ovarian inflammation, Ovulation, Salmeterol

## Abstract

**Background:**

Ovarian chronic inflammation has been known to incidence in the laying hen mainly via increasing laying frequency and microbial infection, especially during late stage of production period. This study was aimed to evaluate beta-2 adrenergic agonist (Beta-2 Adrenergic Agonist, BAA) Salmeterol and beta blocker (Beta Blocker, BB) Propranolol on the gene expression of the ovarian pro- and anti-inflammatory mediators, inflammatory responses of immune system, ovarian functions and, hormones in the laying hens on the late stage of production period. Forty-eight White Leghorn hens aged 92 weeks were used for 4 weeks to be supplemented by Salmeterol and Propranolol. Ovulation rate and follicular growth were determined based on laying frequency and ovarian visual evaluation, respectively; the mRNA expressions of follicular beta-2 adrenergic receptor (Beta-2 Adrenergic Receptor, β2ADR), cyclooxygenases (Cyclooxygenases, COX) 1 and 2, and cytokines were measured by real-time PCR. The plasma concentration of ovarian hormones, cellular, and humoral immune responses were measured via ELISA, heterophil to lymphocyte ratio (Heterophil to Lymphocyte ratio, H:L), and sheep red blood cell (Sheep Red Blood Cell, SRBC) test, respectively.

**Results:**

As compared to control, both of BAA Salmeterol and BB Propranolol resulted in a significant decrease in the mRNA expression of β2ADR, cyclooxygenases, and pro- and anti-inflammatory cytokines (*P* < 0.01). A significant elevation was observed in the ovulation rate (*P* < 0.05), plasma estradiol content on both treated groups (*P* < 0.05), and the content of progesterone and was just significantly (*P* < 0.05) increased in Salmeterol group. H:L was reduced in BAA group (*P* < 0.05), and immunoglobulin (Ig) M was elevated in both treated hens, when compared to control. The results indicated that Salmeterol significantly increases body weight (*P* < 0.05).

**Conclusion:**

The stimulation and inhibition of beta-2 adrenergic signaling could reduce ovarian inflammatory condition in addition to enhancing laying efficiency in the aged laying hens.

## Introduction

For the recent decades, due to the improvement of genetic technologies and breeding schedules, production efficiency has been increased in the farm animals like laying hens. Nevertheless, this improvement has remained some reproductive consequences like ovarian chronic inflammation in the laying hens’ reproductive organs compare to the wild birds and the native laying hens [1, 2]. Besides, the immune system has been indicated to influence ovarian inflammatory condition via the outbreak and intensify of microbial infection and the high frequency of ovulatory process which accompany with infiltration of leukocytes and the production of inflammatory mediators such as cytokines [3–5]. These could be as the justifiable reasons to contribute in the deterioration of production rate and egg quality in the laying hens [6], especially, in the late stage of production period [1].

Approximately during 2 last decades, some evidence reported that the ovarian chronic inflammation was controlled in the aged laying hens by administrating some anti-inflammatory strategies like herbal drugs [7], non-steroidal anti-inflammatory drug (Non-Steroidal Anti-Inflammatory Drug, NSAID) [8] and the sources enriched by Omega-3 fatty acids [9] that all of them improved the ovarian chronic inflammation. Therefore, the presentation and evaluation of different anti-inflammatory strategies may improve the ovarian inflammation in the aged laying hens. Among these, the agonists and blockers of beta-2 adrenergic receptors have been shown to create the anti-inflammatory functions in the different tissues. On one hand, as the anti-inflammatory agents, usage of some beta-2 adrenergic agonists (Beta-2 Adrenergic Agonist, BAA) have been recommended to treat the immune, urinary, nervous, cardiovascular, and respiratory dysfunctions [10–14]. Beta blockers (Beta Blocker, BB), on the other hand, decrease the inflammatory signs in the diseases like rheumatoid arthritis, respiratory disorders, and cancers [15–18]. However, their potential pro-inflammatory role of BAA and BB has been reported on nervous and immune systems in some of researches [19–21].

Therefore, the purpose of this study was to investigate the pro- or anti-inflammatory role of BAA and BB on the inflammatory responses (the mRNA expression of pro-inflammatory cytokines, storied hormones, and functions) in the ovary and immune system of laying hens in the late stage of production.

## Results

### mRNA expression of pro- and anti-inflammatory mediators and β2ADR

**Fig. 1:**
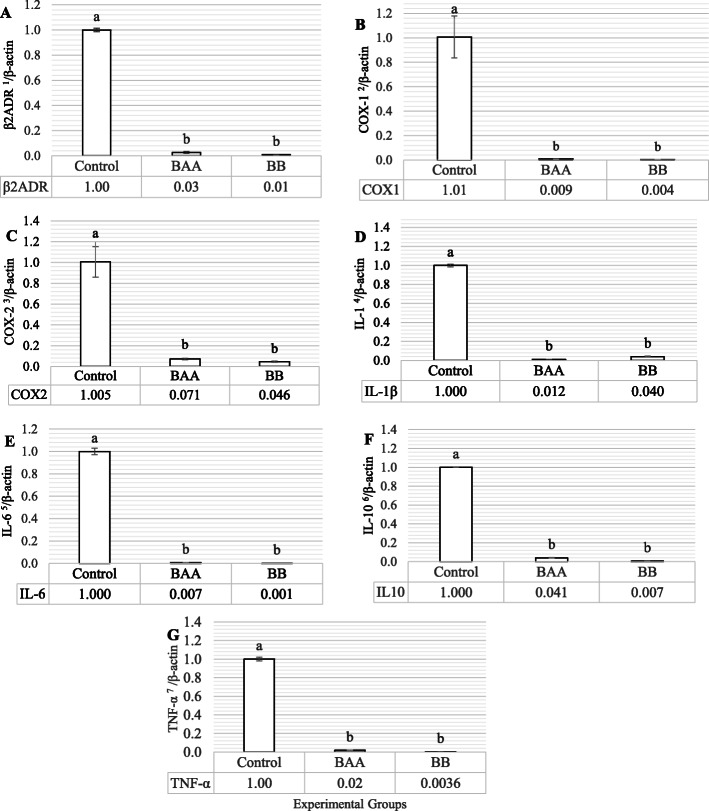
The comparison of β2ADR (**a**), COX-1 (**b**), COX-2 (**c**) IL-1β (**d**), IL-6 (**e**), IL-10 (**f**), and TNF-α (**g**) mRNA expressions between control and treated groups. 1) Beta-2 adrenergic receptor, 2) Cyclooxygenases-1, 3) Cyclooxygenases-2, 4) Interleukin-1β, 5) Interleukin − 6, 6) Interleukin − 10, and 7) Tumor necrosis factor-α. β2ADR, COX-1, COX-2, IL-1β, IL-6, IL-10, and TNF-α mRNA data that  were normalized by β-actin. (BAA) Beta-2 adrenergic agonist (Salmeterol, 1 mg/kg live BW) and (BB) Beta blocker (Propranolol, 2 mg/kg live BW), Different statistical letters (a-c) are significant (*p* < 0.05) according to the Duncan’s multiple range test

The relative abundances of beta-2 adrenergic receptor (Beta-2 Adrenergic Receptor, β2ADR) and cyclooxygenases (Cyclooxygenases, COX) 1 and 2, the cytokines of Interleukin (Interleukin, IL)-1β, IL-6, IL-10, and Tumor Necrosis Factor-α (Tumor Necrosis Factor-α, TNF-α) mRNAs in the pre-ovulatory follicles (pre-ovulatory follicles, F1), normalized to β-actin as a housekeeping gene, were shown in Figs. 1 (a-g). According to Fig. 1. β2ADR, COX-1, COX-2, IL-1β, IL-6, IL-10, and TNF-α expressions were significantly lower in both of BAA and BB compare to the control (*P* < 0.01).

### ELISA analyses of plasma estradiol, progesterone, and androgen

**Fig. 2:**
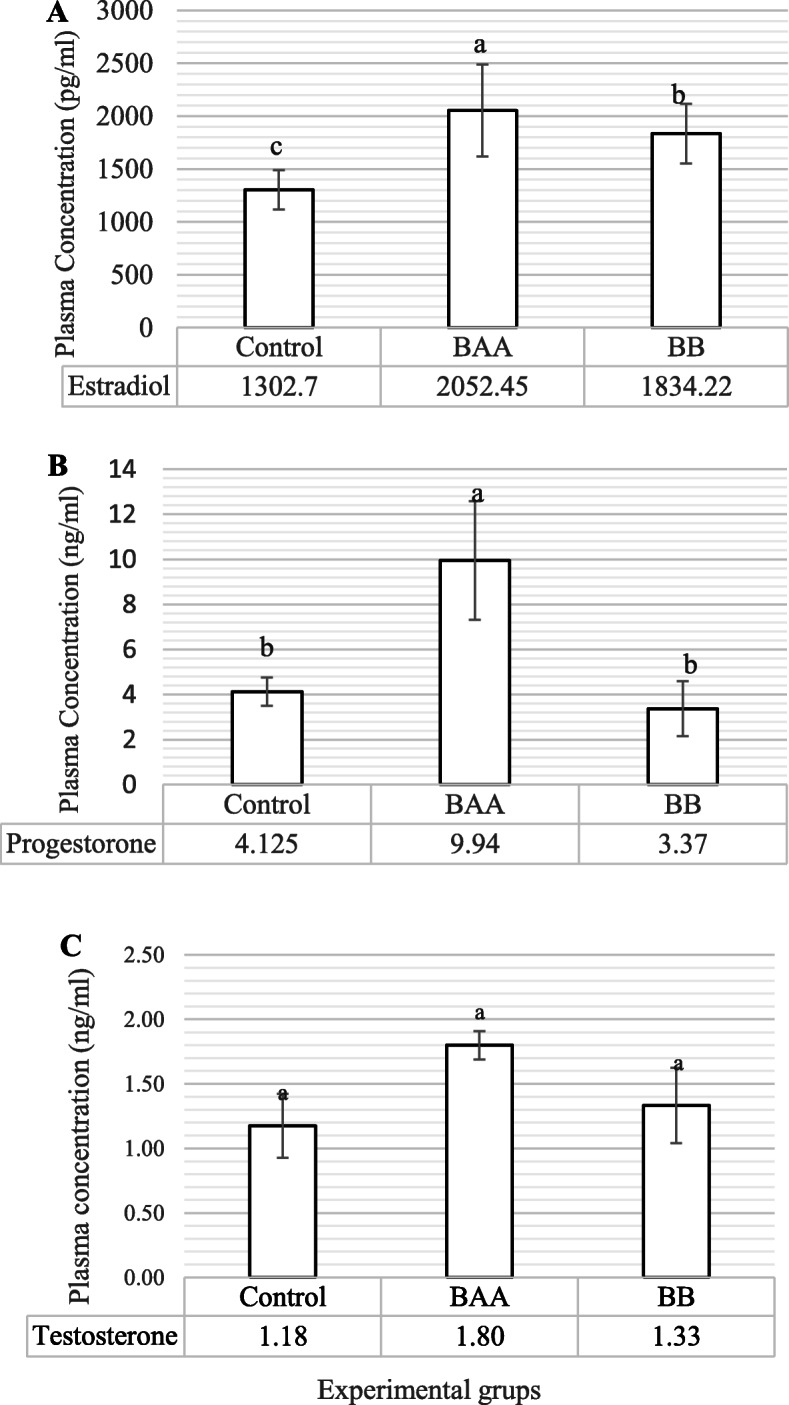
The comparison of plasma Estradiol (**a**), Progesterone (**b**), and Testosterone (**c**) contents between control and treated groups. (BAA) Beta-2 adrenergic agonist (Salmeterol, 1 mg/kg live BW) and (BB) Beta blocker (Propranolol, 2 mg/kg live BW), Different statistical letters (**a**-**c**) are  significant (p < 0.05) according to the Duncan’s multiple range test

Changes in the estradiol, progesterone, and androgen (testosterone) in the plasma contents of control and treated laying hens have been presented in Fig. 2 a-c. Compare to control, the hens supplemented by BAA and BB, significantly had a higher (*P* < 0.01, Fig. 2 a) plasma content of estradiol (*P* < 0.01). Like this hormone, BAA significantly caused to elevate (*P* < 0.01, Fig. 2 b) plasma content of progesterone as compared to control. Plasma content of testosterone was statistically similar (*P* > 0.05, Fig. 2 c) between treated groups and control.

### The function of cellular and humoral immunities

**Fig. 3:**
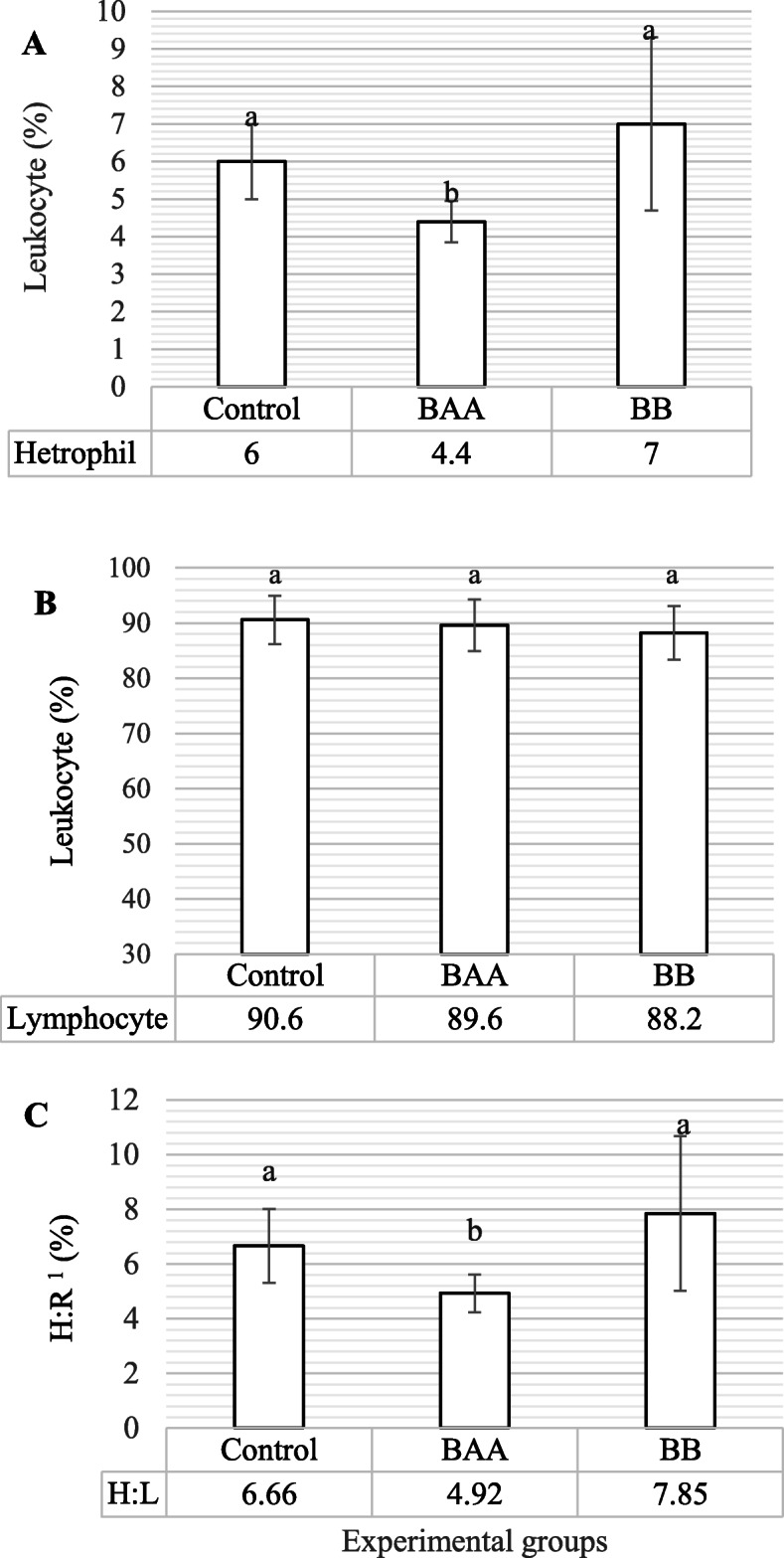
The comparison of heterophil (neutrophil) (**a**), lymphocyte (**b**), and heterophile: lymphocyte ratio (**c**) between control and treated groups. (BAA) Beta-2 adrenergic agonist (Salmeterol, 1 mg/kg live BW) and (BB) Beta blocker (Propranolol, 2 mg/kg live BW), Different statistical letters (a and b) are significant (*p* < 0.05) according to the Duncan’s multiple range test. 1) Heterophil: Lymphocyte ratio

**Fig. 4:**
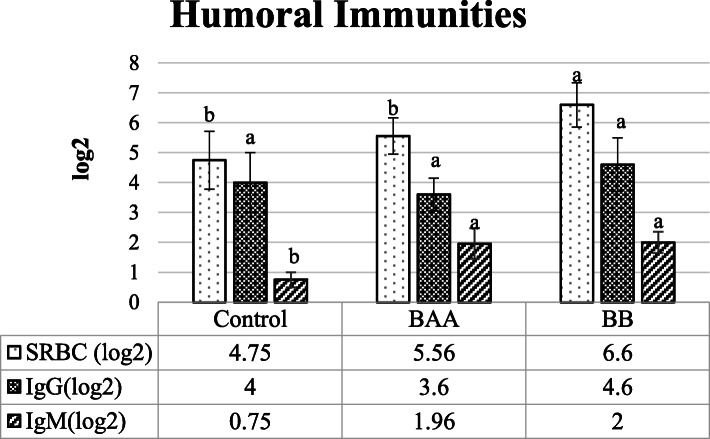
The comparison of whole immunoglobulin (Ig, SRBC), IgG, and IgM contents between control and treated groups. (BAA) Beta-2 adrenergic agonist (Salmeterol, 1 mg/kg live BW), and (BB) Beta blocker (Propranolol, 2 mg/kg live BW), Different statistical letters (a and b) are significant (*p* < 0.05) according to the Duncan’s multiple range test. Anti-SRBC titers were measured and reported as log2 of the last dilution’s reciprocal after the whole agglutination

Figure 3 a-c has shown the changes of neutrophil (heterophil in the avian species), lymphocyte percentages, and heterophil to lymphocyte ratio (Heterophil to Lymphocyte, H:L); and the serum content of immunoglobulins (Immunoglobulin, Ig) G, M, and whole immunoglobulin content (Sheep Red Blood Cells, SRBC) were shown in Fig. 4. According Fig. 3 b, although, there was no significant change in lymphocyte percentage on BAA and BB groups when compared to control (*P* > 0.05), BAA group significantly had the fewer heterophil percentage when compared to control and BB groups (*P* < 0.05, Fig. 3 a). The change of neutrophil percentage observed in BAA group resulted in a significant reduction of H:L (*P* < 0.05, Fig. 3 c) compare to control and BB groups

Despite the non-significant change of IgG between control and treated laying hens’ serum (*P* > 0.05), IgM content was significantly higher in BAA and BB groups than control (*P* < 0.05). Observed changes of IgG and IgM caused to increase (*P* < 0.05) the whole content of Ig (SRBC) in BB group when compared to BAA and control groups.

### Ovarian and body functions

**Fig. 5:**
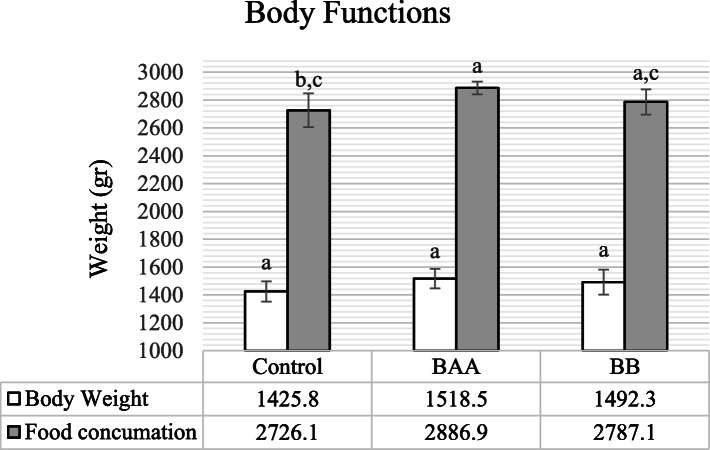
The comparison of body weight and food consummation between control and treated groups. (BAA) Beta-2 adrenergic agonist (Salmeterol, 1 mg/kg live BW) and (BB) Beta blocker (Propranolol, 2 mg/kg live BW), Different statistical letters (a-c) are significant (*p* < 0.05) according to the Duncan’s multiple range test

The changes in hens’ average live body weight (Body Weight, BW) and food consummation as the criteria of body function have been presented in Fig. 5 and their ovulation rate (laying frequency) and follicular sizes F1 to F5 have been shown in Table 1. According to Fig. 5, average BW was significantly similar (*P* < 0.05) between control and treated groups; whereas Food consummation was significantly higher in the BAA group (*P* < 0.05). Results showed in Table 1 that the ovulation rate was significantly increased in BAA (*P* < 0.01) and BB (*P* < 0.05) groups. Moreover, according to this table, there was no significant difference (*P* > 0.05) between BAA and BB groups in follicular sizes F1 to F5 compare to control.

**Table 1:** The comparison of hen’s ovulation rate and follicular sizes F1 to F5 in control and treated groups (mean ± SD)

	Control	BAA^**2**^	BB^**3**^	SEM^**4**^	***p***-value
**Ovulation rate**^**1**^ **(%)**	61.05 ± 8.93^5 b^	72.15 ± 8.89^a^	67.3 ± 9.97^a^	2.9	< 0.05
**Follicular size**					
Follicle F1 (mm)	27.85 ± 5.39^a^	32.4 ± 0.32^a^	30.52 ± 2.24^a^	2.06	0.152
Follicle F2 (mm)	27.25 ± 3.15^a^	27.52 ± 3.05^a^	25.24 ± 6.39^a^	2.05	0.188
Follicle F3 (mm)	20.77 ± 4.4^a^	23.33 ± 4.5^a^	20.08 ± 3.66^a^	1.93	0.7
Follicle F4 (mm)	15.62 ± 4.31^a^	19.37 ± 2.74^a^	15.71 ± 4^a^	1.77	0.44
Follicle F5 (mm)	11.38 ± 2.02^a^	13.43 ± 1.18^a^	9.85 ± 2.11^a^	1.34	0.62

1) Egg laying frequency, 2) Beta-2 adrenergic agonist (Salmeterol, 1 mg/kg live BW), 3) Beta blocker (Propranolol, 2 mg/kg live BW), 4) Standard error of the mean, 5) Standard deviation (SD), and Different statistical letters (a-b) are significant (*p* < 0.05) according to the Duncan’s multiple range test

## Discussion

β2ADR has been reported to enhance the anti-inflammatory properties in some organs and tissues, like immune [11], urinary [12], nervous [13], cardiovascular [14], and respiratory systems [10]. This receptor affects its mentioned functions mainly through activating the canonical signaling pathway β2ADR/ Gs protein (Gs protein, Gs)/cAMP/protein kinase A (Protein kinase A, PKA) [12]. However, this receptor exhibited pro-inflammatory effects by switching from Gs to Gi protein (Gi protein, Gi) which triggers the non-canonical pathways, such as Gi/Phosphoinositide 3-kinase (Phosphoinositide 3-Kinase, PI3K)/ Protein kinase B (Protein kinase B, Akt)/glycogen synthase kinase 3 beta (Glycogen Synthase Kinase 3 beta, GSK3β) and Gi/Ras/Raf/Mitogen-activated protein kinase kinase (Mitogen-activated protein Kinase Kinase, MEK)/extracellular-signal-regulated kinase (Extracellular-signal-Regulated Kinase, ERK) [22]. Therefore, promotion or inhibition of β2ADR activity may play the considerable role to decrease the ovarian and immune inflammatory signs in the aged laying hens in addition to the improvement of their reproductive efficiency.

### Ovarian mRNA expressions

As shown in the Fig. 1 a, compare to control, it was observed the significant mRNA down-regulation of β2ADR in both of BAA and BB groups. Cellular density of β2ADR derives from the various factors like the type of tissue [23, 24], cellular age [25], inflammatory condition [24], and overstimulation of β2ADR for excessive BAA exposure [23]. Concerning the factors mentioned above, observed decrease in β2ADR mRNA expression in this study could probably be as consequence of β2ADR overstimulation BAA Salmeterol (1 mg/kg live BW) and prolonged agonist exposure (4 weeks) in the hens’ ovarian epithelial tissue within the inflammatory condition derived from immune functions and high frequency of ovulation. On the other hand, according to the results attained by the previous studies about the evaluation of BAA Salmeterol on membrane β2ADR density [26, 27], the administration of Salmeterol results in a considerable stabilization of membrane β2ADR because of having a very low efficacy for stimulating β-arrestin and G protein-coupled receptor kinase enzyme (G Protein-coupled Receptor Kinase, GRK) phosphorylation as essential mediators to induce β2ADR desensitization, down-regulation and internalization. Improvement of membrane β2ADR density could be as a main factor to decrease cellular synthesis and degradability turnover of β2ADR proteins and consequently causes to decline its gene transcription. In line with β2ADR mRNA abundance in BAA group, Propranolol resulted in mRNA down-regulation in this receptor that was in agreement with the previous studies that reported a down-regulation after Propranolol treatment [28, 29]. However, BB mainly inhibits β2ADR signaling via receptor β2ADR desensitization, down-regulation, and internalization resulted of GRK/β-arrestin signaling [30].

As the rate-limiting enzymes, cyclooxygenase-1 and 2 (COX) have the critical role in the various physiological roles, and be involved in different ovarian reproduction processes like ovulation [31]. Although, COX-1 is expressed in the majority of cells and tissues and remains in constant expression under most physiologic conditions, COX-2 is inducible and generally only expressed in response to various inflammatory reactions. Cytokines, on the other hand, as products of immune cells, are also synthesized by an extensive range of non-immune cells, like the normal ovarian cells; and their action in the ovary has been described as the motivational processes of follicular development, activation of leukocytes required for ovulation, and tissue remodeling during ovulation [32]. Among these, TNF-α, IL-1β and IL-6 [33] as the pro-inflammatory cytokines, and IL-10 as an anti-inflammatory cytokine [34] play their role in inflammatory reactions. Here, we reported that the supplementation of BAA Salmeterol and BB Propranolol down-regulated COX-1, 2 and pro- and anti-inflammatory cytokines (Fig. 1, b-g). Despite this fact that some studies reported that other BAAs lead to a significant increase in mRNA and protein expressions of three cytokines TNF-α, IL-1β, and IL-6 [35, 36], Salmeterol has been known to inhibit the secretion of these cytokines. In this regard, Hu et al. showed that Salmeterol inhibits the activation of Mitogen-activated protein kinase (Mitogen-Activated Protein Kinase, MAPK) and nuclear factor kappa-light-chain-enhancer of activated B cells (Nuclear Factor Kappa-light-chain-enhancer of activated B cells, NF-ƙB) [37] as main pathways of inducing some pro-inflammatory cytokines including IL-1β, IL-6 and TNF-α [38]. Moreover, according to Shore’s study, TNF-α and IL-1β synergistically perform to promote β2ADR desensitization through the induction of COX-2 expression [39]. Therefore it is believed that the down-regulation of TNF-α and IL-1β, derived from the supplementation of BAA Salmeterol (Fig. 1, d and g), causes not only to decrease COX-2 expression but also to reduce β2ADR desensitization. Anyway, the lower rate of IL-10 mRNA in the BAA group was in contrast to the previous studies that have worked on Salmeterol [40, 41] and the other BAAs [11]. Reduction in COXs and cytokines mRNA expressions in BB group was in line with the previous findings [42–45].

Although BAA and BB benefit from β2ADR signaling pathway with the contradictory functions, they approximately demonstrated the similar results, in particular mRNA expressions of β2ADR, anti-, and pro-inflammatory cytokines. In this regard, Gargiulo et al. clarified the fact that several BB do not really act as pure antagonists and some of them show the same final action to their agonists through the different or similar mechanisms [46]. Additionally, Sozzani et al. suggested that the effect of Propranolol at high doses is not mediated by β2ADR but by its membrane stabilizer properties [47].

### The function of ovarian hormones

β2ADR plays a considerable role in the different ovarian events like ovulation, hormonal secretion, and puberty [48, 49]. As major reproductive steroid hormones, estradiol, progesterone, and testosterone (androgen) play the functional roles to regulate growth, differentiation, and function of an extensive range of target tissues in the females’ reproductive system [50]. However, these hormones have different inflammatory effects. Some evidence confirmed that estrogen demonstrates the dual role depending on the concentration. In the chronic inflammatory diseases, estradiol inhibits important pro-inflammatory cytokines such as TNF, IL-1β, IL-6 at high levels; whereas, these cytokines are stimulated at lower concentrations of estradiol [51]. Progesterone, on the other hand, has a protective role to prevent from inflammation during pregnancy by reducing IL-6 and TNF-α, and by the recovery of antioxidant enzyme performance in some tissues [52]. Androgen therapy reduces the inflammatory process and declines the intensity of disease by mechanisms which inhibit inflammatory cytokines expression and function like TNF-α, IL-1β, and IL-6 [53]. Unlike mammals, hens do not form corpus luteum, and their progesterone is produced by granulosa cells in mature follicles and reaches maximum concentration, approximately, 4–6 h before the ovulation, like estradiol that is produced by theca externa layer [54, 55]. Besides, progesterone, in the birds, is a substantial storied for the pathway of estradiol and testosterone production [56]; therefore, the change of progesterone concentration influences the plasma content of these hormones. About the results expressed in Fig. 2 a-c, the birds supplemented by BAA and BB, significantly had a higher plasma estradiol content, as compared to control. Plasma progesterone content was higher in the BAA group than BB and control. In keeping with our results, the previous evidence reported that catecholamines elevated plasma estradiol, progesterone, and androgen concentrations in the experimental animals [57–59]. This up-regulation not only is derived from theca layer stimulation of the ovarian follicles [57] but also has is influenced by indirect regulation of the pituitary gonadotrophs response to Gonadotropin-releasing hormone (Gonadotropin-Releasing Hormone, GnRH) [60] that these routes are activated through prevalent beta-2 adrenergic signaling of formation of cAMP. Whereas, GnRH release is down-regulated via intracellular cAMP signaling which is blocked by Propranolol [61]. Therefore, lower mRNA expression of ovarian pro-inflammatory cytokines in this study could derive from the anti-inflammatory behavior at higher concentrations of estradiol and progesterone in BAA and BB groups.

### The function of cellular and humoral immunities

The immune inflammatory markers like H:L have been mentioned as a considerable index of the systemic inflammatory response for predicting the prognosis of different diseases with inflammatory origin [62]. Generally, the factors that increase inflammatory signs, were accompanied to higher H:L, and factors decreasing inflammation, were associated lower H:L [63]. The elevation of H:L is created via increasing circulating heterophils and decreasing lymphocytes counts. Our results demonstrated in Fig. 3 (a-c) that the administration of BAA Salmeterol significantly caused a reduction in neutrophil percentage and H:L as compared to BB and control groups. These results were in agreement with some studies that demonstrated H:L could be as independent and straightforward predictor for inflammation-originated respiratory disorders like asthma and chronic obstructive pulmonary disease (Chronic Obstructive Pulmonary Disease, COPD) [64] that are treated by BAA [65]. In this regard, some documents have shown that activation of the β2ADR inhibits inflammatory responses in neutrophils via the various intra-cellular pathways like clearance of cytosolic Ca2^+^, inhibition of the generation of superoxide anion (O2(•-)) production [66, 67], and release of acetylcholine that exerts its anti-inflammatory effects binding to alpha-7 nicotinic receptors [68].

As components involved in anti-inflammatory reactions, immunoglobulins contribute to attract other immune cells on sites of inflammation, facilitate the anti-inflammatory processes, and prevent inflammatory reactions [69]. According to the main autoantibodies, IgG and IgM were found wide clinical application as anti-inflammatory agents in various inflammatory and autoimmune diseases [70, 71]. Figure 4 shows that despite significantly having the similar serum content of IgG between BAA and BB groups, serum content of IgM was higher in these treatments compare to control that these finding were in line with the previous studies [72, 73]. In this regard, Sanders 2012 mentioned that the activation of two pathways LynCD19/Akt/NF-ƙB/p50/p65 and PLCγ0032α/ Protein kinase C (Protein Kinase C, PKC)/p65 which play the role of increase in the amount of IgG1 per B cell, were found to converge by cAMP response element-binding protein (cAMP Response Element-Binding protein, CREB) as a down-stream compound of beta-2 adrenergic signaling pathway [74].

### Ovarian and body functions

Ovulation is defined as inflammatory phenomenon which has been approved by two hypotheses incessant ovulation (Fathalla’s incessant ovulation hypothesis) and inflammation [2]. Fathalla has theorized the continuous involvement of the ovarian surface in the ovulatory process because of incessant processes rupture and repairing of the wound on the ovarian surface. Over time, these processes boost the ovarian chronic inflammation. On the other hand, according to the inflammation hypothesis, the ovulation-related events have been reported to resemble an inflammatory reaction that accompany with leukocytes infiltration and production of inflammatory mediators like cytokines, vascular endothelial growth factor (Vascular Endothelial Growth Factor, VEGF), prostaglandins, and intracellular signaling pathways closely associated with inflammatory reaction [75]. Regarding the results shown in Table 1, the laying hens, supplemented by BAA Salmeterol and BB Propranolol, significantly indicated more ovulation rate and the similar follicle size F1 to F5 as compared to control that was in agreement with the studies which showed catecholamines and Propranolol improve the ovulation rate and follicular development [76, 77]. Besides, Fig. 5 demonstrated that food consummation was elevated in the BAA group, and BW was similar between BAA and BB groups in comparison with control. In addition to the influence of inflammatory events, the factors like nutritional-metabolic factors and relevant hormones of the hypothalamus-pituitary-ovary axis play the fundamental roles in the functions of ovulation and follicular development. About the effect of nutritional-metabolic factors, some evidence demonstrated energy balance, nutrients (fatty acids, glucose, and amino acids), and metabolic hormones like insulin, insulin-like growth factor 1 (Insulin-like Growth Factor 1, IGF-I), and growth hormone implicate in ovarian functions such as the follicular development and ovulation [78]. Increase in food intake, observed in the BAA group, not only caused to improve live BW that represents positive energy balance but also confirmed as one of the reasons [78] for increasing ovulation rate. Moreover, BAA was shown to increase insulin, IGF-I [79, 80], and growth hormone [81] which promote ovulation and follicular growth. Whereas, Propranolol was reported to decrease insulin and IGF-1 and increase growth hormone [82–84]. GnRH, gonadotropins, and ovarian hormones, on the other hand, act as preliminary effects on follicular development and ovulation [85]. For these reasons, enhanced ovulation rate could also be as results of elevated plasma estradiol and progesterone, and increase in food intake in the birds administrated by BAA Salmeterol and increased plasma estradiol in BB group. Therefore, as one of the contributing factors of ovulation, pro-inflammatory mediators which their mRNA expressions were down-regulated in the BAA and BB Groups, do not seem to have enough capability on ovulation rate in these groups in comparison to the effects of ovarian hormones and metabolic status.

## Conclusion

The results of this study have indicated that the administration beta-2 adrenergic agonist (BAA) Salmeterol and beta blocker (BB) Propranolol caused to down-regulate mRNA expressions of the pro-inflammatory mediators and beta-2 adrenergic receptor. Salmeterol and Propranolol could create an anti-inflammatory condition via increasing some of ovarian hormones and decreasing the inflammatory criteria of immune system. Despite reduction in pro-inflammatory factors in ovary, ovulation rate increased in the hens treated by Salmeterol and Propranolol because of better nutritional status and ovarian hormones situation in these groups. Taken together, both strategies of stimulating and inhibiting beta-2 adrenergic signaling are capable of reducing ovarian inflammatory condition in addition to increase in laying efficiency in the late stage of production period of commercial laying hens.

## Materials and methods

### Animal care

Forty eight 92-week-old commercial strains of White Leghorn laying hens (*Gallus domesticus*) were housed at the poultry research farm, department of animal sciences, University of Tehran at Karaj. Laying hen husbandry was adjusted and approved by the institutional animal care of this institute. The birds were exposed to a photoperiod of 16 h light: 8 h dark with lights on at 06:00 and lights off at 22:00, food and water provided ad libitum. As body function, laying frequency (ovulation rate) and feed intake were monitored and recorded. The value and ingredients of the test diet were indicated in Table 2.

**Table 2:** The Ingredients (%) and nutrient composition of the diet

Diets	Value (%)
Corn	61.00
Soybean meal	23.45
Sodium bicarbonate	0.05
D-calcium phosphate	1.53
fatty acid	2.81
Salt	0.07
Calcium carbonate	10.47
Vitamins + Minerals	0.50
DL-methionine	0.13
**Calculated analysis**	
Crude protein	15.39
Calcium	4.62
Available phosphorus	0.40
Metabolizable energy	2780^a^

^a^ (kcal/kg)

All laying hens were randomly divided and orally supplemented into three groups (*n* = 16) included: control, BAA Salmeterol (1 mg/kg live Body Weight, BW), and BB Propranolol (2 mg/kg live BW) for 4 weeks. Supplemented levels of Salmeterol (Jaber Ebne Hayyan Pharma. Co., Tehran, Iran) and Propranolol (Mehr Darou Pharma. Co., Tehran, Iran) mentioned above, had previously been obtained by a pre-trial according production efficiency.

### Blood collection

For evaluating cellular and humoral immunities and ovarian hormones responses, blood samples (5.0 mL/hen) were randomly collected from the brachial vein of 8 laying hens per group at the end of 4 weeks and their centrifuged serum and plasma (at 3000 rpm for 15 min) were stored at -20o C for determination of humoral immune and ovarian hormones, respectively.

### Immune responses

Blood samples were smeared on to a glass slide to calculate of the heterophil to lymphocyte ratio (H:L) as an inflammatory criterion of cellular immunity. After drying, the smears were stained with May-Grünwald-Giemsa stain [86]. The H:L was calculated by dividing the number of heterophils by the number of lymphocytes. For measuring humoral immunity, on the 14th and 20th day of the experiment, all of hens were injected with 0.1 mL of 0.25% suspension of sheep red blood cells (sheep red blood cells, SRBC, provided from a healthy male sheep) in phosphate buffer saline. Anti-SRBC antibody titers of hens’ serum were obtained by the micro hemagglutination technique from samples taken from blood collection at the end of the experiment. Anti-SRBC titers were measured and reported as log2 of the last dilution’s reciprocal after the whole agglutination [87].

### Ovarian hormones measurement

The levels of plasma hormones of estradiol, progesterone, and testosterone were determined in this study by ELISA kits (Monobind® Inc., USA), given the mentioned manufacturer’s recommendations. The sensitivity of detection, intra-, and inter-assay coefficients of variation (%) for estradiol were 6.5 pg/mL, 6.3, and 8.5%, for progesterone were 0.105 ng/mL, 1.5% and below 13% and for testosterone were 0.038 ng/mL, 4.9, and 4.6%, respectively.

### Tissue sampling

After four weeks, 10 hens per experimental group were euthanized by CO_2_ asphyxiation and necropsied. In this step, ovaries were removed and their yellow follicles were arranged base on their diameter (from F1 as pre-ovulatory follicles to F5 as 5th small yellow follicle) measured from follicle stigma. After measuring follicle size, Pre-ovulatory follicles (12–35 mm) were removed from ovaries, washed by saline, kept at microtube, and stored at − 80 °C for RNA isolation.

### RNA isolation and cDNA synthesis

Total cellular RNA was isolated from frozen tissues using Trizol reagent (RNX-plus, Cinagen Co., Tehran, Iran) according to the manufacturers’ recommendations. The quantity and quality of total RNA were determined by spectrometry and denaturing agarose gel electrophoresis, respectively. For RNA purification, samples were treated with DNase I (YT 9054, Yekta Tajhiz Azma co., Tehran, Iran) before reverse transcription reaction. cDNA was synthesized by the cDNA reverse transcription kit (YT4500, Yekta Tajhiz Azma co., Tehran, Iran). The obtained cDNA was stored at − 80 °C for analyzing gene expression using real-time PCR [31].

### Real-time PCR

Target gene mRNA levels were measured using SYBR green qPCR master mix (YT 2550, Yekta Tajhiz Azma co., Tehran, Iran) and a real-time rotary analyzer (Rotor-Gene 3000, Corbet Research, USA). Hen specific primers were gathered in Table 3. β-actin was used as housekeeping gene to normalize target gene expression. Amplification conditions: 95 °C for 300 s followed by 50 cycles of 95 °C for 10 s and 60 °C for 30 s with melt curve measured at 65–95 °C every 0.5 °C gradient for 5 s. Control reactions lacking template were run for each target gene. Reactions were 10 μL in total volume and 200 nM of each primer. The relative levels of mRNA expression were analyzed by the 2^-ΔΔC^_T_ method [88].

**Table 3:** Chicken primers used for real-time PCR

Gene	Accession No.	primers sequences (5′ → 3′)	Orientation
β2ADR ^1^	XM_004950587	GACGCCGGAACGCTGAG	Forward
		GAAGACAGTGACCAGCACGA	Reverse
COX-1 ^2^	XM_425326	TCAGGTGGTTCTGGGACATCA	Forward
		TGTAGCCGTACTGGGAGTTGAA	Reverse
COX-2 ^3^	XM_422297	CTGCTCCCTCCCATGTCAGA	Forward
		CACGTGAAGAATTCCGGTGTT	Reverse
IL-1β ^4^	AB559570	CTTCCTCCAGCCAGAAAGT	Forward
		CAGCTTGTAGCCCTTGAT	Reverse
IL-6 ^5^	AB559572	CAACCTCAACCTGCCCAA	Forward
		GGAGAGCTTCCTCAGGCATT	Reverse
IL-10 ^6^	AB559574	CACAACTTCTTCACCTGCGAG	Forward
		CATGGCTTTGTAGATCCCGTTC	Reverse
TNF-α ^7^	AY765397	TGTGTATGTGCAGCAACCCGTAGT	Forward
		GGCATTGCAATTTGGACAGAAGT	Reverse
β-Actin ^8^	L08165	CATCACCATTGGCAATGAGAGG	Forward
		GCAAGCAGGAGTACGATGAATC	Reverse

1) Beta-2 adrenergic receptor, 2) Cyclooxygenases-1, 3) Cyclooxygenases-2, 4) Interleukin-1β, 5) Interleukin − 6, 6) Interleukin − 10, and 7) Tumor necrosis factor-α. 8) β2ADR, COX-1, COX-2, IL-1β, IL-6, IL-10, and TNF-α mRNA data that are normalized by β-actin

### Statistical analysis

According to general linear model (general linear model, GLM), data were analyzed and compared by Duncan multiple range test using SPSS software (IBM SPSS Statistics, version 26.0, 2019). Statistical significance of each parameter was considered as significant at *P* ≤ 0.05.

## Data Availability

The data used or analyzed are all included in this published article.

## Abbreviations

Akt: Protein kinase B
BAA: Beta-2 adrenergic agonist
BB: Beta blocker
BW: Body weight
β2ADR: Beta-2 adrenergic receptor
COPD: Chronic obstructive pulmonary disease
COX: Cyclooxygenases
CREB: cAMP response element-binding protein
ELISA: Enzyme-linked immune sorbent assays
ERK: Extracellular-signal-regulated kinase
F1: Pre-ovulatory follicles
GLM: General linear model
GnRH: Gonadotropin-releasing hormone
Gi: Protein
Gs: Gs protein
GRK: G protein-coupled receptor kinase
GSK3β: Glycogen synthase kinase 3 beta
H:L: Hetrophil to lymphocyte ratio
Ig: Immunoglobulin
IGF-I: Insulin-like growth factor 1
IL: Interleukin
MAPK: Mitogen-activated protein kinase
MEK: Mitogen-activated protein kinase kinase
NF-ƙB: Nuclear factor kappa-light-chain-enhancer of activated B cells
NSAIDs: Non-steroidal anti-inflammatory drug
PKA: Protein kinase A
PKC: Protein kinase C
PI3K: Phosphoinositide 3-kinase
SRBC: Sheep red blood cell
TNF-α: Tumor necrosis factor
VEGF: Vascular endothelial growth factor
